# ImmuneRegulation: a web-based tool for identifying human immune regulatory elements

**DOI:** 10.1093/nar/gkz450

**Published:** 2019-05-22

**Authors:** Selim Kalayci, Myvizhi Esai Selvan, Irene Ramos, Chris Cotsapas, Eva Harris, Eun-Young Kim, Ruth R Montgomery, Gregory Poland, Bali Pulendran, John S Tsang, Robert J Klein, Zeynep H Gümüş

**Affiliations:** 1 Department of Genetics and Genomic Sciences, Icahn School of Medicine at Mount Sinai, New York, NY 10029, USA; 2 Icahn Institute for Data Science and Genomic Technology, Icahn School of Medicine at Mount Sinai, New York, NY 10029, USA; 3 Department of Microbiology and Global Health and Emerging Pathogens Institute, Icahn School of Medicine at Mount Sinai, New York, NY 10029, USA; 4 Department of Neurology, Yale University, New Haven, CT 06510, USA; 5 Division of Infectious Diseases and Vaccinology, School of Public Health, University of California, Berkeley, CA 94720, USA; 6 Division of Infectious Diseases, Department of Medicine, Northwestern University Feinberg School of Medicine, Chicago, IL 60611, USA; 7 Section of Rheumatology, Department of Internal Medicine, Yale School of Medicine, New Haven, CT 06520, USA; 8 Mayo Clinic, 200 First St SW Rochester, MN 55905, USA; 9 Emory Vaccine Center/Yerkes National Primate Research Center at Emory University, Atlanta, GA 30329, USA; 10 Multiscale Biology Section, Laboratory of Immune System Biology, National Institute of Allergy and Infectious Diseases, NIH, Bethesda, MD 20892, USA; 11 NIH Center for Human Immunology, Bethesda, MD 20892, USA

## Abstract

Humans vary considerably both in their baseline and activated immune phenotypes. We developed a user-friendly open-access web portal, ImmuneRegulation, that enables users to interactively explore immune regulatory elements that drive cell-type or cohort-specific gene expression levels. ImmuneRegulation currently provides the largest centrally integrated resource on human transcriptome regulation across whole blood and blood cell types, including (i) ∼43,000 genotyped individuals with associated gene expression data from ∼51,000 experiments, yielding genetic variant-gene expression associations on ∼220 million eQTLs; (ii) 14 million transcription factor (TF)-binding region hits extracted from 1945 ChIP-seq studies; and (iii) the latest GWAS catalog with 67,230 published variant-trait associations. Users can interactively explore associations between queried gene(s) and their regulators (cis-eQTLs, trans-eQTLs or TFs) across multiple cohorts and studies. These regulators may explain genotype-dependent gene expression variations and be critical in selecting the ideal cohorts or cell types for follow-up studies or in developing predictive models. Overall, ImmuneRegulation significantly lowers the barriers between complex immune regulation data and researchers who want rapid, intuitive and high-quality access to the effects of regulatory elements on gene expression in multiple studies to empower investigators in translating these rich data into biological insights and clinical applications, and is freely available at https://immuneregulation.mssm.edu.

## INTRODUCTION

Recent high-throughput studies are contributing to an improved understanding of immune cell function and regulation (1). Extensive datasets are now available through the Human Immunology Project Consortium (HIPC) Phase 1, which includes measurements of baseline and activated human immune system, coupled with detailed clinical phenotyping in well-characterized cohorts (2–5). HIPC Phase 2 is continuing to collect data and expand the available datasets. In addition, large collaborative studies, including the Genotype-Tissue Expression Project (GTEx) (6), Framingham Heart Study (7) and Encyclopedia of DNA Elements (ENCODE) (8–10), are collectively generating thousands of sequencing-based, genome-wide measurements of the transcriptome, transcription regulatory regions, transcription factor binding, and others that define states of the genome in many cell types and tissues, including a significant number of immune cell types. Furthermore, the NHGRI-EBI GWAS catalog (11) provides a centralized summary of large-scale studies that yield associations between genetic variants and various traits.

The wealth of data generated by these studies can be utilized for a deeper understanding of the mechanisms by which gene expression is regulated in different immune cells and immune systems of individuals in steady state and in response to immune stimuli (e.g. pathogens, disease, and vaccines). Genetic variants typically appear to localize in the regulatory regions of genes and alter gene expression levels both proximally (putatively cis-acting) and distally (putatively trans-acting) (12), while transcription factors tend to bind directly to promoter regions proximally upstream of a gene, or directly to the RNA polymerase molecule and alter gene expression (13). However, often in the discovery phase, it is difficult to assess the real biological role of these regulatory elements without extensive experimentation on a large number of humans or immune cell types. While several studies that have identified cis- and trans-acting variants on gene expression have made their results publicly available for download and/or provided web interfaces, these typically include text-only displays (14–18). To take advantage of the already generated rich datasets in the context of metadata that accompany them, a user-friendly immune-specific platform is needed to integrate, visualize and interactively explore these datasets. Such a platform can support investigations on human immunity, infection and disease as well as in the development of predictive models and therapies.

We have built an interactive web-based visual interface, ImmuneRegulation, which capitalizes on the existing knowledge of the associations between transcription regulatory elements and gene expression changes in well-defined cohorts. ImmuneRegulation enables the exploration of massive immune-system specific gene regulation datasets and provides a centralized repository with seamless integration of information gained from large publicly available dataset resources to help efforts in understanding the associations between the immune system and its regulation. Simple visual queries to interact with these datasets assist discovery by creating resources useful for hypothesis generation. Users can simultaneously query multiple resources to identify cell-type or cohort specific regulatory elements that drive the expression of genes or gene sets of interest; study potential inherited susceptibility to specific responses or disease; or design follow-up cohort response studies with improved sensitivity and accuracy of classification by excluding or including certain individuals with specific genetic variants.

ImmuneRegulation currently includes genetic variant-gene expression associations on ∼220 million expression quantitative trait loci (eQTLs). These enable the identification of gene(s) whose expression are affected by human germline genetic variation in blood or blood cell types. Additionally, transcription factor (TF) datasets from publicly available ENCODE Consortium ChIP-seq studies in humans are included. Furthermore, GWAS catalog data within ImmuneRegulation repository provide information about published SNP-trait associations.

In summary, ImmuneRegulation is a unique resource focused on the regulation of gene(s) that are phenotype-dependent in expression response to immune stimuli. More specifically, it is an interactive web-based visual tool that enables the exploration of massive data relevant to the regulation of immune-system specific genes in well-defined cohorts. Furthermore, ImmuneRegulation is currently the only visual interface for exploration of trans-eQTLs. Prior to ImmuneRegulation, these datasets were publicly available in separate resources without any exploratory features. Overall, ImmuneRegulation provides a centralized repository that enables seamless integration of information gained from HIPC studies with public dataset resources to help efforts in understanding the associations between the immune system and its regulation, where users can capture regulatory elements that drive some of the phenotypic differences observed in immune-related transcriptomes. ImmuneRegulation is available at https://immuneregulation.mssm.edu.

## MATERIALS AND METHODS

**Figure 1.**
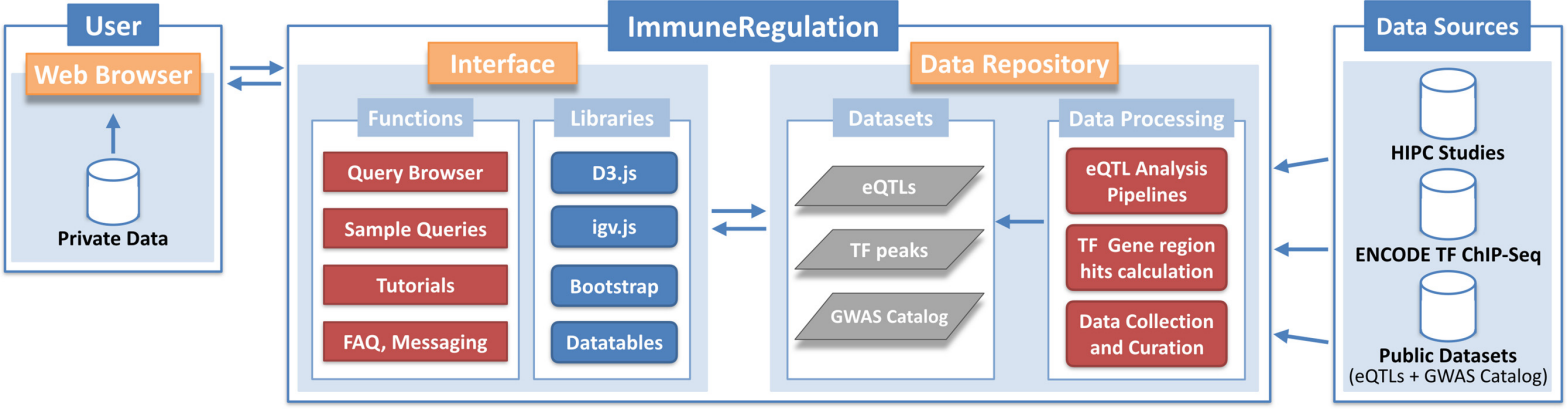
ImmuneRegulation overall architecture and main components.

We provide a schematic of the ImmuneRegulation interface architecture and main components in Figure 1. As shown, we built the ImmuneRegulation data repository from a curated collection of multiple sources, on which we performed different levels and types of processing, depending on the data type. The full list of data sources currently available within ImmuneRegulation data repository is provided in Supplementary Table S1. Data repository and interface design details are provided below.

### Data collection and processing

#### Publicly available eQTL datasets

We curated a collection of publicly available datasets from whole blood or blood cell type studies to make them available within the ImmuneRegulation repository (full list, including data sources, is in Supplementary Table S1).

#### cis- and trans-eQTLs from HIPC genotype-gene expression datasets

Next, we have identified eQTLs from all HIPC datasets where both genotype and gene expression data were available. These currently include (i) systemic genotype and gene expression array profiling of influenza-vaccinated individuals at multiple time points, and (ii) RNA-seq profiling of individuals at both acute and convalescent stages of Chikungunya virus infection. All datasets are listed in Supplementary Table S1. For studies that include both genotype and gene expression data from the same individuals on array platforms, we downloaded pre-processed gene expression datasets in tab-delimited text format from ImmuneSpace (www.immunespace.org) and the genotype data in Plink binary PED format from studies generated as part of HIPC Phase I and II. For the Chikungunya virus infection study, we downloaded and processed raw RNA-seq data from NCBI GEO database (accession GSE99992) for variant calling and the pre-processed RNA-seq data from (19) for gene expression analysis. To discover short variants from raw RNA-seq data, we first aligned spliced reads to human genome GRCh37 using STAR 2-pass (20), and then performed SNP and indel calling using the Genome Analysis Toolkit (21) (GATK, https://www.broadinstitute.org/gatk/) best practices workflow.

To identify significant eQTLs from either array or RNA-seq platform datasets, we utilized the eQTL mapping pipeline provided at https://github.com/molgenis/systemsgenetics/wiki/eQTL-mapping-analysis-cookbook-(eQTLGen). For cis-eQTL analysis, we followed steps 1–6 in the pipeline. For trans-eQTL analysis, we continued with step 8 in which we regressed out the effects of cis-eQTLs obtained in the cis-eQTL mapping step. Specifically, these steps include population stratification using Multi Dimensional Scaling (MDS) of genotype data using Plink v1.07 (22); harmonization of genotype files using Genotype Harmonizer v1.4.9 (23); and removal of expression outliers, normalization of gene expression data, identification of sample mix-ups between genotype and gene expression data, calculation and removal of principal components that are not under genetic control, and cis-eQTL and trans-eQTL mapping, using eqtl-mapping-pipeline tool v1.2.4 (https://molgenis50.gcc.rug.nl/jenkins/job/systemsgenetics/271/nl.systemsgenetics$eqtl-mapping-pipeline/). We added all new cis- and trans-eQTLs we identified in these studies with FDR ≤ 0.05 to ImmuneRegulation data repository.

#### Transcription Factors (TF) datasets

We downloaded all human TF datasets from the publicly available ENCODE Consortium ChIP-seq studies (https://www.encodeproject.org, downloaded in April 2018; this dataset will be updated once every 6 months) and incorporated into ImmuneRegulation data repository (24,25). These include 1945 ChIP-seq TF-binding site datasets (in bed narrowPeak file format) from 144 different cell/tissue types and 736 different TF targets. For each dataset, we calculated gene region hits using bedtools v2.25.0 (26), and associated each gene with the metadata of all relevant datasets that contain gene region hit(s). These metadata include information on data tracks (i.e. ChIP-seq peak files) that are then transferred from https://www.encodeproject.org upon need.

#### GWAS datasets

We have curated and incorporated the complete NHGRI-EBI GWAS catalog (11) (www.ebi.ac.uk/gwas, downloaded in March 2018; this dataset will be updated once every 6 months) into ImmuneRegulation data repository. Currently, this includes information about 67,230 published SNP-trait associations.

### Implementation

#### Implementation

ImmuneRegulation utilizes multiple client-side Javascript libraries (e.g. D3.js, JQuery, etc.) to facilitate visualization and user interaction with large volumes of multiple data types in real time. For visualizations that are displayed within the genome browser, we incorporated igv.js (https://igv.org/doc/doc.html) and customized it to better serve the specific needs of our tool. We also incorporated several other utility libraries (e.g. dataTables.js, underscore.js, etc.) for data manipulation and interaction. For web interface styling, we primarily relied on Bootstrap v3.3.7, integrated with our custom CSS elements. Since our implementation utilizes only standard libraries and does not necessitate any external plug-ins, ImmuneRegulation runs on all modern web browsers. At the back-end of ImmuneRegulation, each cis-eQTL dataset is compressed and indexed using Tabix (27), whereas trans-eQTL datasets and the GWAS catalog dataset are stored in tab-delimited text files. Meta-data associated with TF datasets are stored in JSON files and the specific TF datasets are fetched from https://www.encodeproject.org at run-time.

## RESULTS

ImmuneRegulation provides a unified platform for immune-specific gene expression regulation datasets in multiple cell types and large human cohorts, in the context of their rich metadata (see Figure 1). In its front-end, a visually intuitive web interface enables query, browsing and interaction with large volumes of data. Users can query for regulatory elements (eQTLs and TFs) of gene(s) of interest in real-time. For gene(s) queried, visual, interactive summaries of regulatory elements are returned to help explore, contextualize and communicate statistical analyses and results. Resulting graphics can be downloaded in pdf, svg, or png format and the associated eQTL data can be downloaded as text files.

### Data repository

Since gene expression regulation is tissue-specific, ImmuneRegulation is an immune system-focused resource to understand regulation in whole blood or blood cells (e.g. T-cells, B-cells, and monocytes). Its data repository currently includes (i) genotype data from more than 43,000 individuals, associated with gene expression data from more than 51,000 experiments, providing genetic variant-gene expression associations on around 220 million eQTLs; (ii) 1945 ChIP-seq TF-binding site datasets associated with 144 human cell/tissue types and 736 TF targets from ENCODE Consortium, where we have extracted 14 million gene region hits from these ChIP-seq datasets and included them as meta-data for exploration at the gene level; and (iii) GWAS catalog data on about 67,230 published SNP-trait associations.

### Getting started (performing queries)

**Figure 2.**
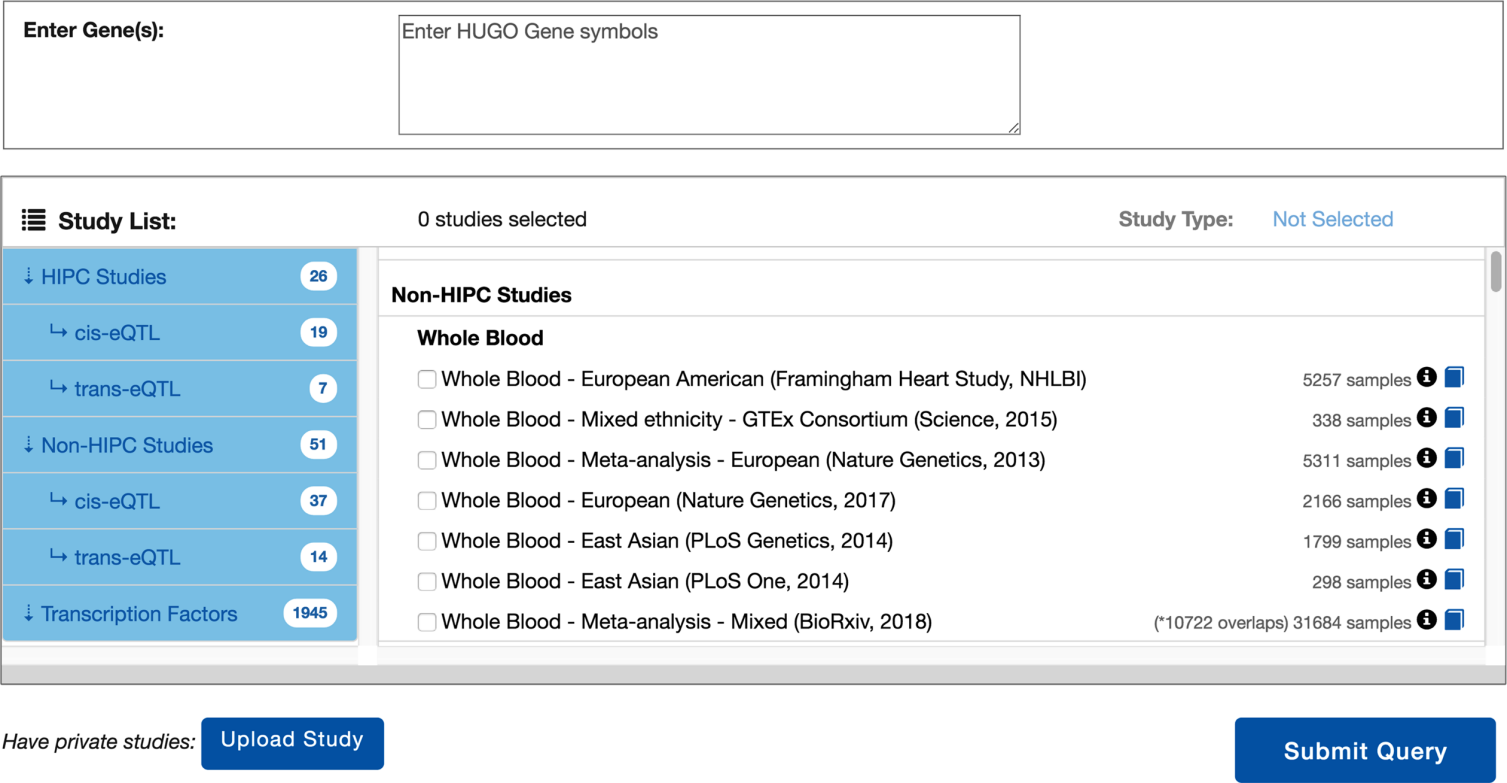
Main visual interface for listing datasets and constructing queries.

The landing page of ImmuneRegulation (Figure 2) provides the main interface for browsing existing datasets and constructing queries. Users can quickly browse the summary information on each dataset (e.g. cell type, cohort size, ethnicity), and, if they need more information on a study, click on the provided links for relevant study details. Users can construct queries by listing their gene(s) of interest in the query box (using HUGO symbols), selecting study types (cis-eQTL, trans-eQTL, TF), and dataset(s) to query, and then clicking on the Submit Query button. Users can then easily check or modify their queries by using the Modify Query button.

#### Querying for cis-eQTLs

**Figure 3.**
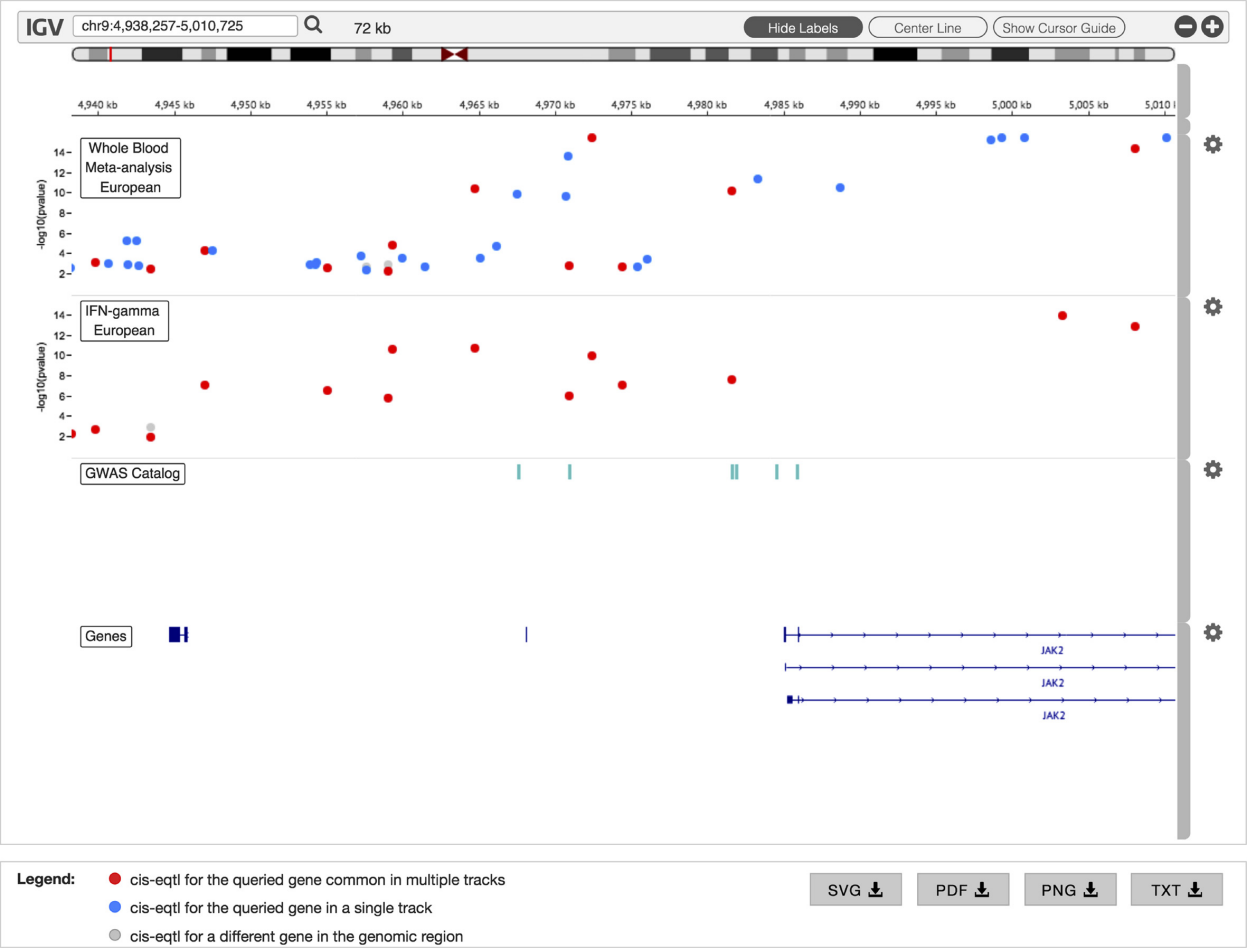
cis-eQTL results for two different datasets displayed within the customized IGV browser. GWAS Catalog and Genes tracks are also displayed by default.

To perform a cis-eQTL query, users enter gene name(s) and select one or more relevant datasets. Users can view results of one gene at a time; to view the results of a different gene, users simply click on its name in the gene list provided. This opens up a customized Integrative Genomics Viewer (IGV) browser (28), where cis-eQTLs and their associated *P*-values are displayed in separate genome browser tracks for each selected dataset (see Figure 3). Here, each cis-eQTL is represented as a circle: blue if it appears in only a single study; red if it appears in multiple studies and gray if it is within the query region, but has been shown to regulate a gene other than the query gene. Clicking on any cis-eQTL circle reveals a pop-up dialog box that displays more details associated with it (position, gene, *P*-value, SNP ID and information link). Two additional data tracks are also displayed within the genome browser to further assist in data exploration. First of these is a GWAS Catalog track, which displays any SNP (represented as a square) within the genomic search region that is associated with a GWAS finding. Clicking on such a SNP opens a pop-up dialog box that displays additional information (e.g. disease/trait, *P*-value, publication, etc.). Second is a gene track, which displays the genomic locations of the query gene and all its isoforms to enable their quick comparison with respect to the relative locations of the regulatory elements within the genome browser.

#### Querying for trans-eQTLs

**Figure 4.**
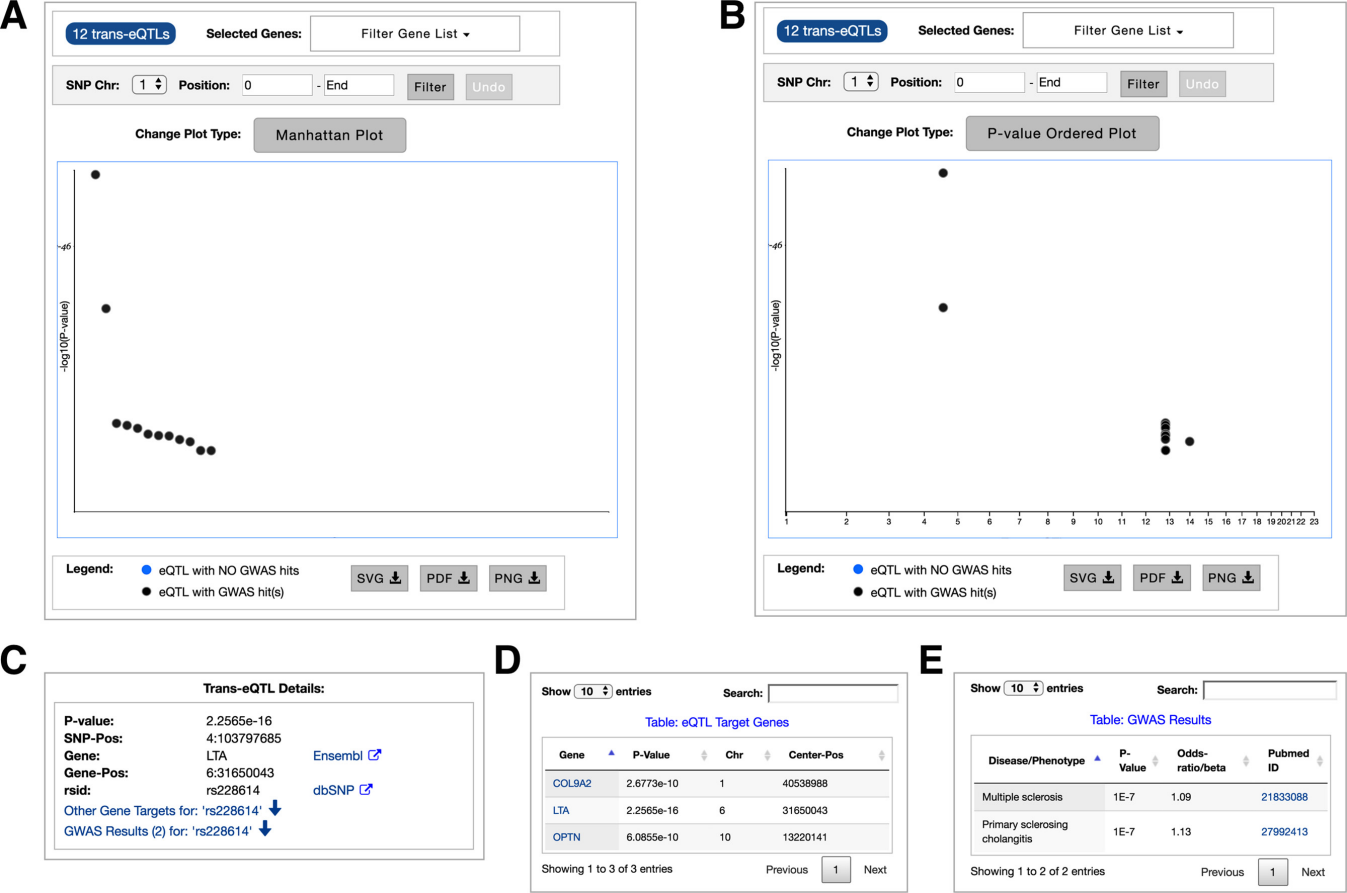
All trans-eQTL results from associated query (**A**) *P*-value ordered; (**B**) Manhattan plot; For a specific trans-eQTL (**C**) details panel; (**D**) eQTL target genes table; (**E**) GWAS results table.

To perform a trans-eQTL query, users enter gene name(s) and select one or more relevant datasets. In multi-gene queries, users can query for trans-eQTLs that regulate their either intersection or union. If intersection option is selected, an interactive table lists all trans-eQTLs that regulate at least two genes in the query gene list. If the union option is selected, or if only a single gene is queried, an interactive graph displays all trans-eQTLs that regulate the query gene(s), either rank-ordered and positioned based on their *P*-values (lowest *P*-value on top) (Figure 4, panel A), or within a Manhattan plot (Figure 4, panel B). Users can further filter these results for a subset of genes or SNP locations. In this interactive graph, black circles represent trans-eQTLs that also have associated GWAS hits, while blue circles represent trans-eQTLs without any such hits. Hovering over any circle provides a quick look into its details via a tooltip text. For deeper explorations, users can click on any trans-eQTL circle to display more information (e.g. *P*-value, SNP ID, etc.) in a separate panel (Figure 4, panel C), which can be further interactively explored to generate tables of other gene targets (panel D) and GWAS hits associated with that specific trans-eQTL (panel E).

#### Querying for TFs

**Figure 5.**
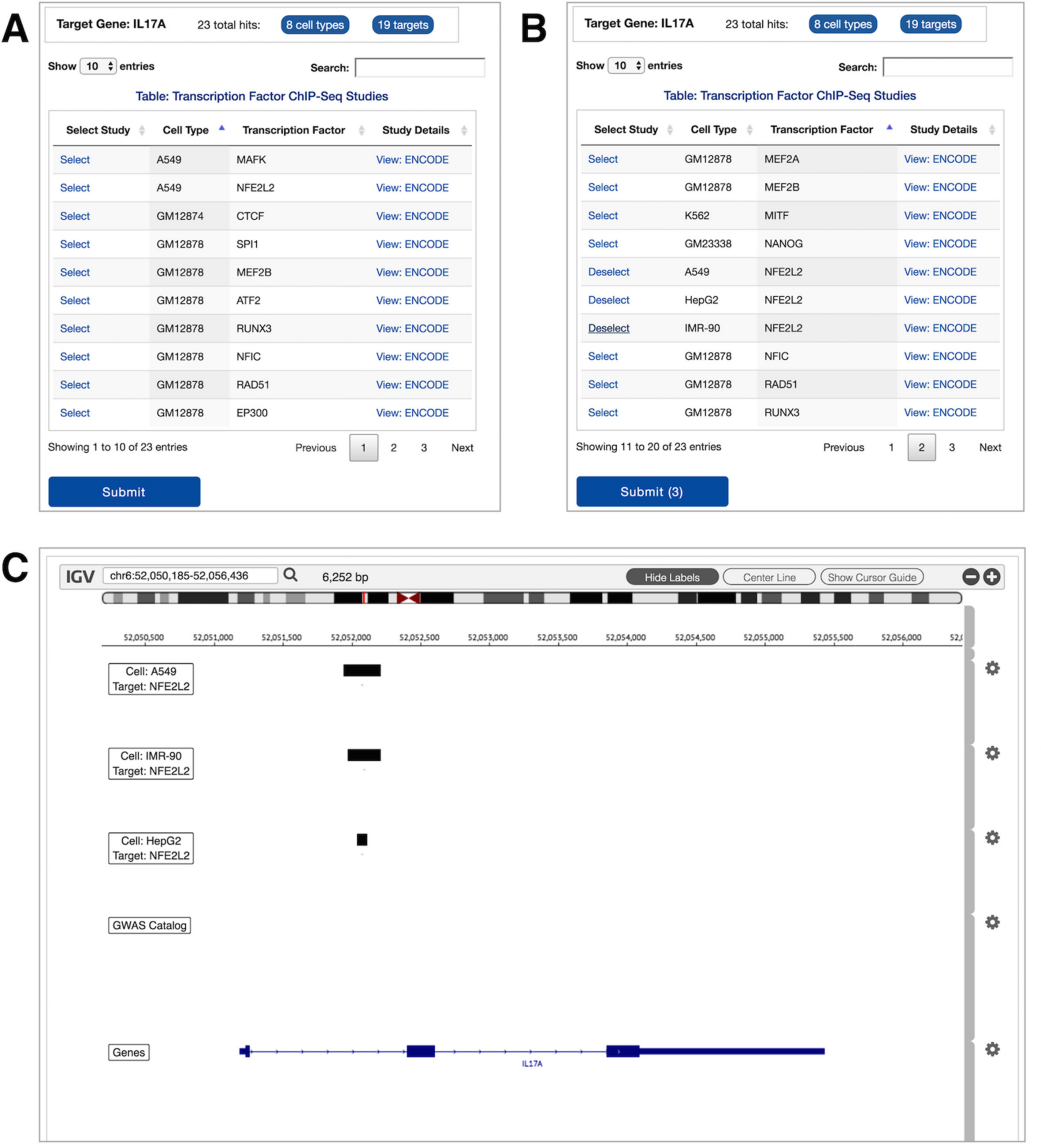
Transcription Factor gene hits results (**A**) table sorted by cell type; (**B**) table sorted by TF, and studies are selected for submission; (**C**) associated ChIP-seq studies loaded and displayed within the IGV browser.

To perform a TF query, users enter gene name(s) and select ENCODE Consortium ChIP-seq datasets. Users can view results of one gene at a time; to view the results of a different gene, users simply click on its name in the gene list provided. This opens an interactive table that lists all datasets that have at least one hit (peak) in the corresponding gene region (Figure 5, panel A). Each row corresponds to a specific cell type-TF combination. Users can query this table for cell types or TFs using the Search box to explore and select datasets (panel B). Clicking on Submit button loads our customized IGV browser that displays the query results (ChIP-seq peaks) (panel C). In addition, GWAS catalog and genes tracks are displayed by default to provide further information on regulators within this genomic region.

### Uploading and visually exploring private data

**Figure 6.**
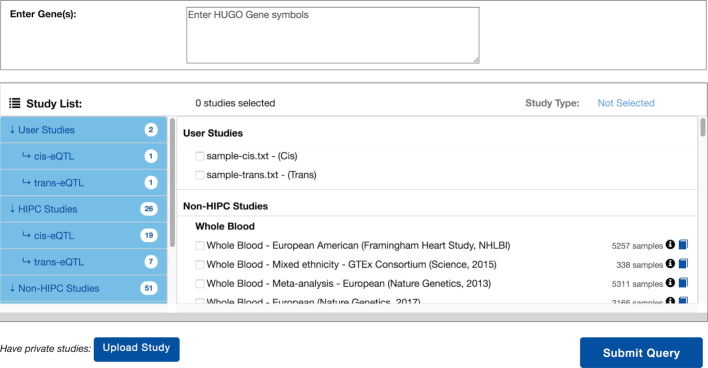
Query interface displaying user uploaded datasets.

ImmuneRegulation provides the capability for users to visualize their own eQTL datasets for integrated analyses with datasets within the interface. An important point here is that the user datasets are not transferred to any remote server or third-party site; all operations are handled completely within the local browser of the user. This functionality can be utilized by clicking on the Upload Study button in the main query browser interface. A pop-up dialog window opens up that includes guidelines to properly format and upload dataset(s). Upon successful completion of data upload, user’s query browser interface looks as in Figure 6. These datasets become completely integrated with the ImmuneRegulation interface; users can query them in combination with existing datasets, and explore the associated results in the same way.

### User interface/ feedback

Since ImmuneRegulation aims to provide a user friendly and intuitive interface, we incorporated various mechanisms for easy user queries and data exploration. First, warning messages are displayed dynamically to help users construct the queries properly. Second, we provide multiple curated queries within the portal for users to gain familiarity with the tool and to easily create customized queries of their own. Third, we provide multiple detailed tutorials that help explain various components and functionalities of ImmuneRegulation. Fourth, an FAQ page provides basic information pertaining to the content as well as technical aspects of the interface (e.g. missing data, load time, etc.). Finally, users can provide feedback or ask questions regarding any technical or scientific matters directly within the ImmuneRegulation interface.

## DISCUSSION

To comprehensively understand heterogeneity in the healthy human immune system and in response to stimuli, multiple groups are generating high-throughput measurements of gene regulation in the human immune system. These massive data create their own challenges in accurately interpreting immunophenotyping data, necessitating easy to use data mining and interpretation tools. An important goal in these efforts is phenotyping the immune states and diseases for diagnosis, prognosis and selection of therapies. ImmuneRegulation helps investigators interpret multiple data resources in their efforts to generate hypotheses on how transcriptome differences observed in different members of the same cohort can be explained by specific regulators. While such regulators (genetic variants or transcription factors) can yield insights into the immune response to stimuli, and can improve sensitivity and accuracy of classification, often in the discovery phase it is difficult to assess their real biological role without extensive experimentation. To help support investigations on human immunity, infection and disease, multiple datasets with existing genotype and transcriptome data on a large number of humans and immune cell types can be integrated in a single, user-friendly interface. The availability of reliably curated patient cohorts and the integrative interface of ImmuneRegulation provides for the exploration of massive immune regulatory datasets that can help accelerate our understanding of the human immune system. We anticipate that rapid, intuitive and integrated analyses within our interface will significantly empower immune researchers in their study of the complex role genomic regulation plays in immune phenotype responses and assist in translating the findings into insights and applications in basic and clinical immunology. Specifically, some of the differences observed in the phenotypic responses may be caused by inter-individual genetic variation, and identifying the regulatory elements that lead to differences in transcriptome responses within these cohorts can yield insights into the development, remission and exacerbation of disease. Identification of these regulatory elements may as well improve diagnostic sensitivity and accuracy of cohort classification, and ultimately guide treatment.

## DATA AVAILABILITY

ImmuneRegulation is an open source collaborative initiative available in the GitHub repository https://github.com/gumuslab-mssm/immuneRegulation

## Supplementary Material

gkz450_Supplemental_Files

## Notes

Present address: Irene Ramos, Department of Neurology, Icahn School of Medicine at Mount Sinai, New York, NY 10029, USA.

Present address: Bali Pulendran, Institute for Immunity, Transplantation and Infection, Department of Pathology, Department of Microbiology and Immunology, Stanford University, Stanford, CA 94305, USA.
