# Supplementary material for: ImmuneRegulation: a web-based tool for identifying human immune regulatory elements
Source: Nucleic Acids Res. 2019 May 22;47(W1):W142–50. doi: 10.1093/nar/gkz450 (PMC6602512; doi:10.1093/nar/gkz450)
Supplement: gkz450_Supplemental_Files [file gkz450_supplemental_files.zip › Supplementary Figures-ALL.pdf]

SUPPLEMENTARY FIGURES

Supplementary Figure 1. cis- and trans-eQTLs identified in the Mayo HIPC study on Influenza vaccine response (FDR ≤ 0.05) (ImmuneSpace ID: SDY67)

Supplementary Figure 2. cis- and trans-eQTLs identified in the NIH HIPC study on Influenza vaccine response (FDR ≤ 0.05) (ImmuneSpace ID: SDY80)

Supplementary Figure 3. cis- and trans-eQTLs identified in the Yale HIPC study on Influenza vaccine response (FDR ≤ 0.05) (ImmuneSpace ID: SDY400, SDY404)

Supplementary Figure 4. cis- and trans-eQTLs identified in the Emory HIPC study on Influenza vaccine response (FDR ≤ 0.05) (ImmuneSpace ID: SDY56)

Mayo HIPC Study – ImmuneSpace ID: SDY67

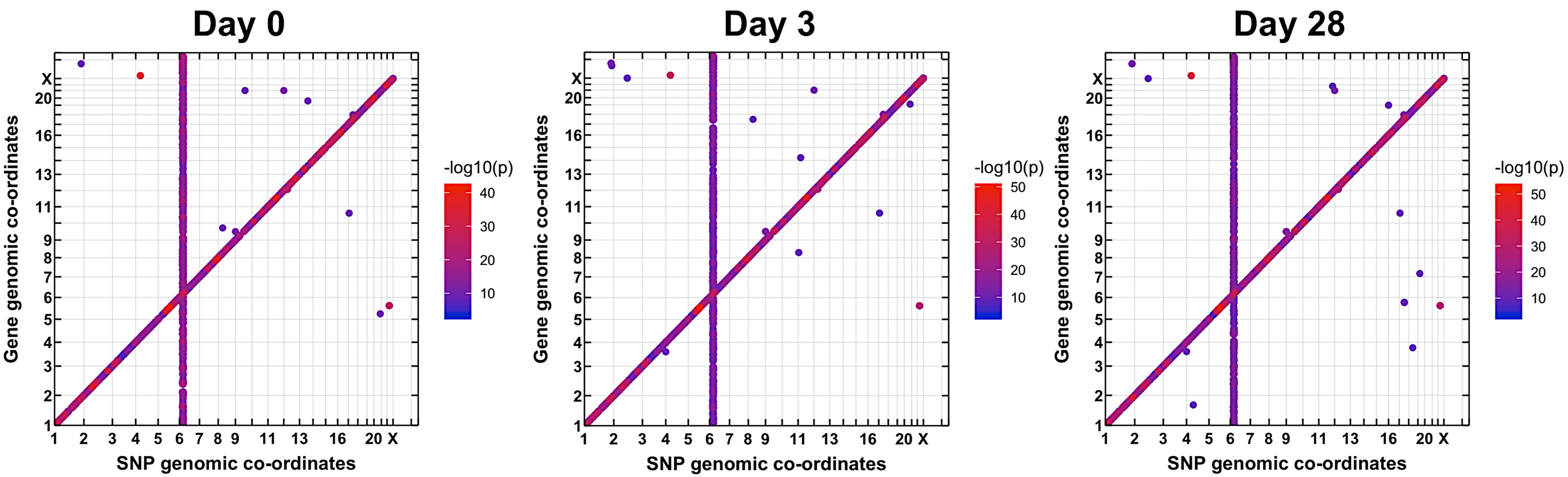

Supplementary Figure 1. cis- and trans-eQTLs identified in the Mayo HIPC study on Influenza vaccine response (FDR ≤ 0.05) (ImmuneSpace ID: SDY67)

NIH HIPC Study – ImmuneSpace ID: SDY80

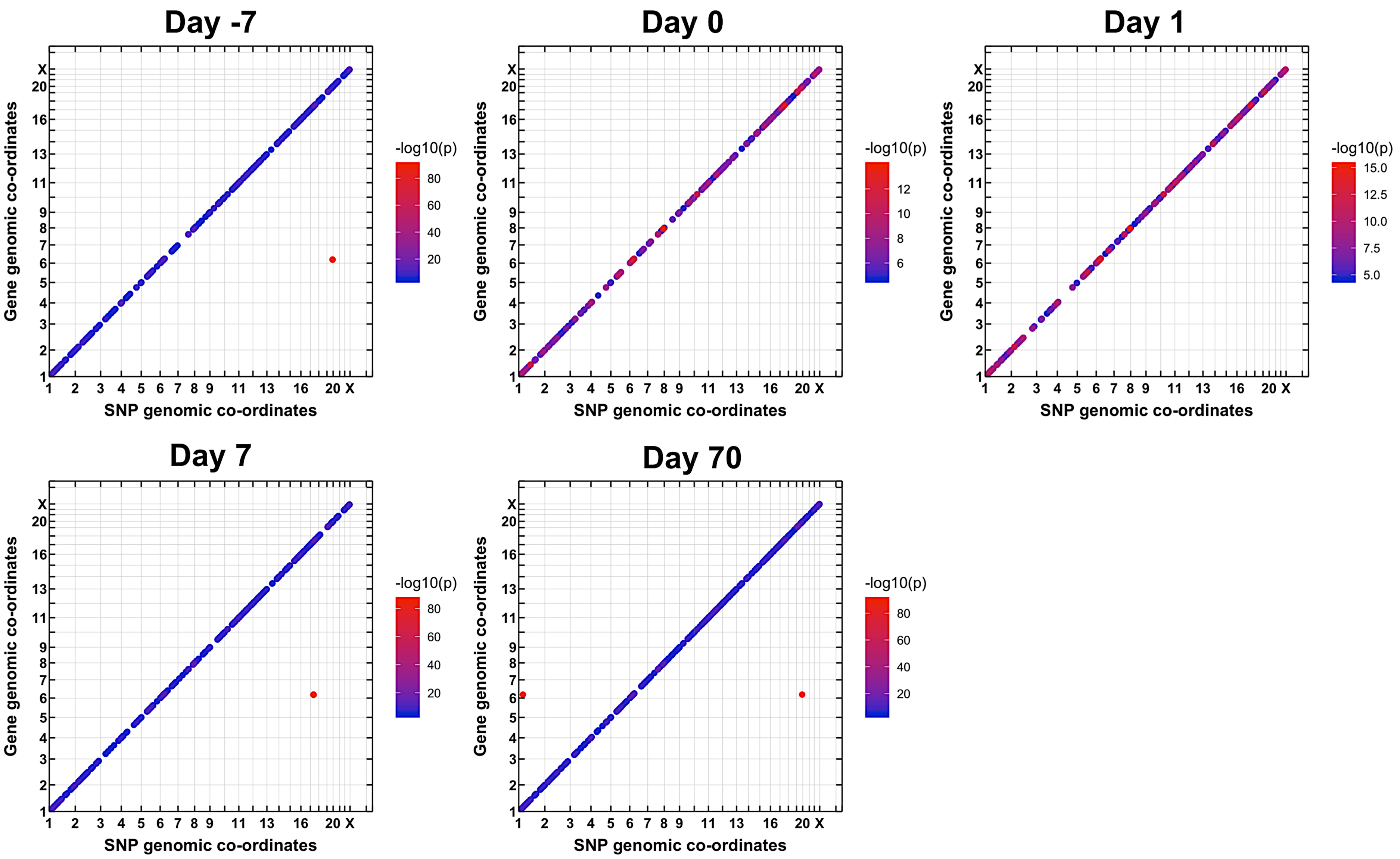

Supplementary Figure 2. cis- and trans-eQTLs identified in the NIH HIPC study on Influenza vaccine response (FDR ≤ 0.05) (ImmuneSpace ID: SDY80)

Yale HIPC Study – ImmuneSpace ID: SDY400, SDY404

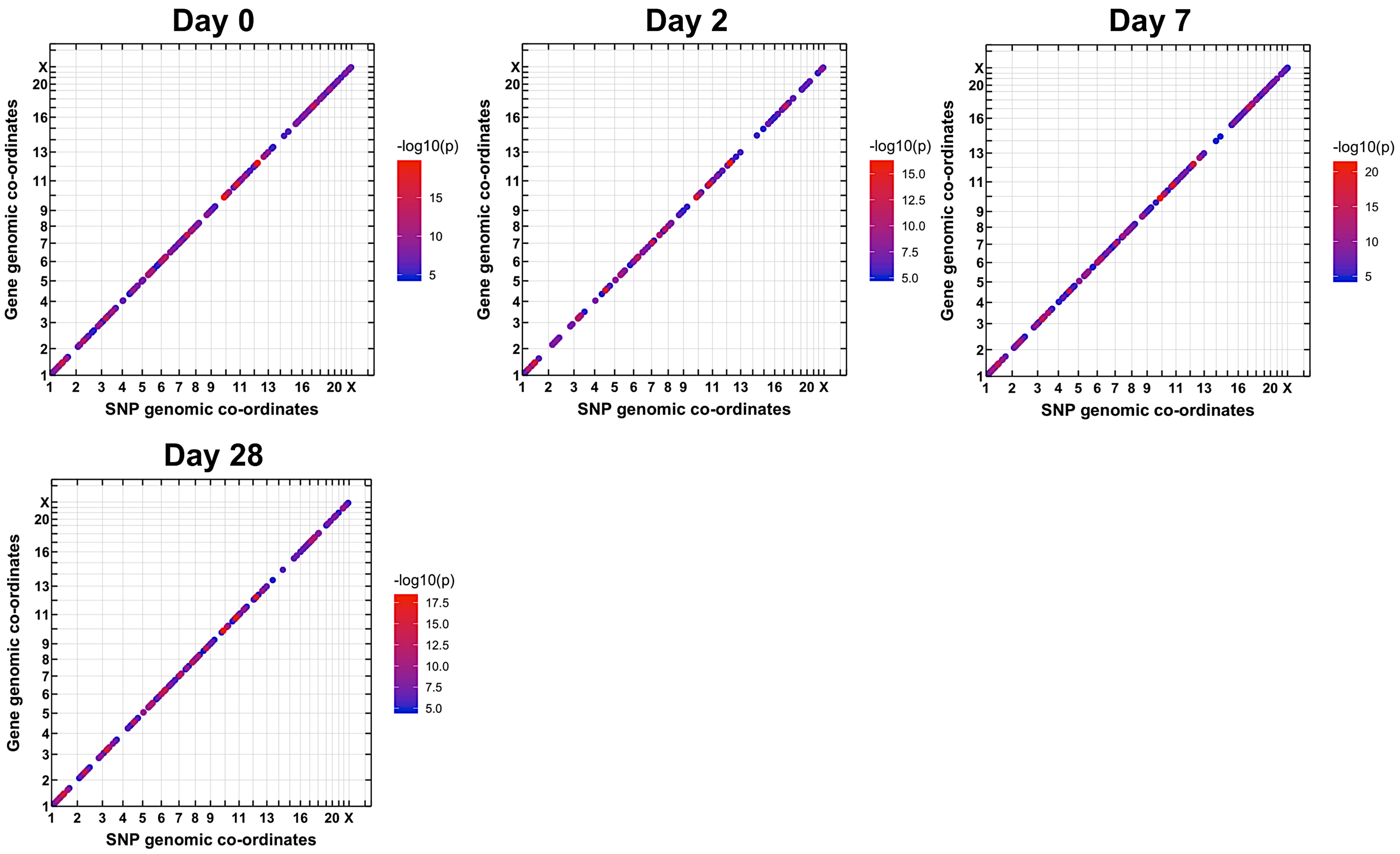

Supplementary Figure 3. cis- and trans-eQTLs identified in the Yale HIPC study on Influenza vaccine response (FDR ≤ 0.05) (ImmuneSpace ID: SDY400, SDY404)

Emory HIPC Study – ImmuneSpace ID: SDY56

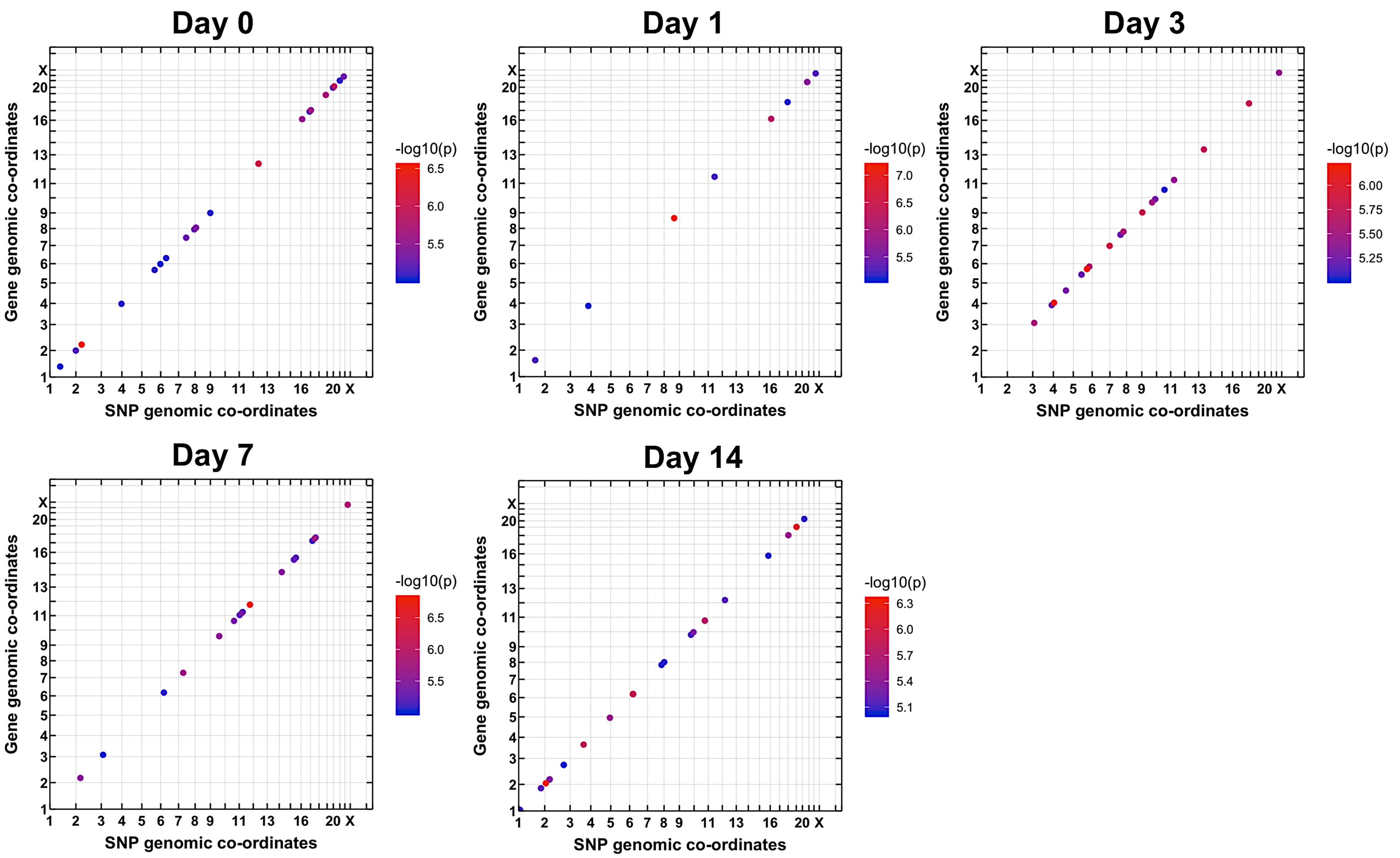

Supplementary Figure 4. cis- and trans-eQTLs identified in the Emory HIPC study on Influenza vaccine response (FDR ≤ 0.05) (ImmuneSpace ID: SDY56)
